# How to promote changes in primary care? The Florentine experience of the House of Community

**DOI:** 10.3389/fpubh.2023.1216814

**Published:** 2023-09-05

**Authors:** Chiara Milani, Giulia Naldini, Lorenzo Baggiani, Marco Nerattini, Guglielmo Bonaccorsi

**Affiliations:** ^1^Department of Health Science, University of Florence, Florence, Italy; ^2^Department of District Healthcare Network, Azienda USL Toscana Centro, Florence, Italy; ^3^Health Society of Florence, Florence, Italy

**Keywords:** primary health care (MeSH), House of Health, House of Community, collaborative practice, Participatory Action Research (PAR), interprofessional education (IPE)

## Abstract

Primary care (PC) has a central role in promoting health and preventing diseases, even during health emergencies. The COVID-19 pandemic has shown how strengthening comprehensive primary healthcare (c-PHC) services is key to ensuring community health. The Italian government decided to support PHC by investing resources from the Next Generation EU (NextGenEu) plan in the development of local health districts (LHDs) and local PC centers called “Houses of Community (HoC)”. The Florence LHD (Tuscany)—in direct collaboration with the University of Florence—has represented the experimental context in which a c-PHC-inspired organizational model has been proposed and included the HoC as the nearest access point to PC services. Through multiprofessional collaboration practices, HoCs provide continuity of care as well as health and social integration. Different levels of action must coexist to initiate, implement, and sustain this new PC model: the organizational and managerial level, the experimentation of a new model of care, and the research level, which includes universities and LHD through participatory research and action approaches. This process benefits from health professionals' (HPs) participation and continuous assessment, the care for working relationships between HPs and services, an appropriate research methodology together with a “permeable” multidisciplinary research group, and educational programs. In this context, the HoC assumes the role of a permanent laboratory of experimentation in PC, supporting the effectiveness of care and answering what the Next Gen EU plan has been foreseeing for the rethinking of Italian territorial services.

## 1. Introduction

The COVID-19 pandemic revealed worldwide the inadequacies of healthcare systems mainly centered on hospital acute care, underlining the urgency to strengthen primary care (1). International organizations have been highlighting the central role of primary care in coping with health emergencies (HEs) (2), suggesting the importance of preserving the innovation of primary care services introduced in response to the COVID-19 pandemic (1). Further evidence supports the crucial role of a strong primary care system in ensuring effective and more appropriate answers to health needs in HE (3–5). Therefore, there is a clear indication toward experimenting with innovative models of providing health promotion and healthcare in community and primary care settings. Despite the strength of the evidence on where to move and how to implement direction, vision, and strong ethical standing, these proofs are clashing with current healthcare management and the system as a whole, that is, the many obstacles to new forms of working organization, daily difficulties, and healthcare professionals' medical education (6–8).

However, examples of primary care centers inspired by the principles of c-PHC have developed in several countries in the pre-pandemic period, both in European contexts, such as Spain (9), Portugal (10), and Italy (11), and in non-European contexts, such as Iran (12) and China (13). The different experiences reported reflect the characteristics of different contexts.

In response to the changing health needs that emerged during the COVID-19 pandemic and in accordance with international evidence, the Italian Government defined a new model of primary care that improves the local health district (LHD) by introducing the House of Community (HoC).

The following subsections contain the theoretical background, the national legislative references, and the elements of the organizational local context in which the process described happens.

## 2. Subsections

### 2.1. Theoretical background (c-PHC)

According to the Alma Ata Declaration, *comprehensive Primary Healthcare (c-PHC)* addresses the main community health issues and involves all health-related sectors to achieve health equity, community, and individual participation in health promotion, and fight against health inequalities (14–16). It foresees the creation of a multiprofessional and multidimensional healthcare network, and the adoption of a model centered on comprehensive promotional, preventive, curative, and rehabilitative care, founded on actions addressed to the social determinants of health, intersectoral approach, and community participation. It aims to ensure the continuity of care by delivering people-centered and integrated care services (17) and providing intersectoral interventions (6). Interprofessional collaboration (IPC) is a recognized core element in taking care of complex health needs and an important and meaningful educational experience for healthcare professionals (HPs) and students (18–20).

This theoretical background represents the guiding principle of primary care strengthening in our local context. The Sections 2.2 and 2.3 introduce the elements of the primary care model in the Italian context and in the local context covered by the study.

### 2.2. Italian context

The Italian government is nowadays supporting the strengthening of primary care through the development of a new reference model of local primary care centers called the “House of Community” (HoC). The HoCs, realized within the public Local Health District (LHD), represent an evolution of the already existent *Casa della Salute (House of Health—HoH)*, a type of primary care structure unevenly developed among Italian regions. The model of HoH aimed to further develop multidisciplinary collaboration, social and health service integration, continuity of care, and community involvement. Indeed, the Italian Government has recently decided to allocate funds from the Next Generation EU (NextGenEu) plan—the recovery plan designed and launched by the EU to emerge stronger from the pandemic—to specifically implement the HoC all over the Italian territory. The recent Italian Minister of Health Decree 77/2022 adopted the NextGenEu indications for the development of a new primary care organization. It introduced an innovative design of the LHD and described the HoC as a direct expression of a community-oriented model centered on the person, their social and family networks, and their living places. Therefore, the LHD constitutes the complex primary care services network in which the HoC represents the foundation of the new (functional and structural) model aimed at reinforcing the role of the community in the health system.

### 2.3. Local context: the LHD of Florence

The LHD of Florence—in the Italian Tuscany region—has been representing the site of a c-PHC-inspired model of primary care, supported by a specific reorganization analysis. In this model, the network of services within the HoCs (evolving from HoHs) is thought to ensure proximity and ease of access to the healthcare system. In fact, the HoCs act as decentralized centers for the management of public health services, located in the different neighborhoods of the city.

In this perspective and approach, HoCs (evolving from HoH) represent the nearest access point for the community to primary care services in the LHD. They provide welcome and service orientation, continuity of care, and social integration. To achieve these purposes, a multiprofessional team—composed of General Practitioners (GPs), nurses, and social workers, coordinated by public health doctors—interacts mainly with the other HPs and services of the HoC (medical specialists, administrative staff, physical therapists, mental health professionals, counseling center, vaccination center, nutrition service, addiction health service) and other LHD services. In this context, the activities of the multiprofessional team include the following:

- Weekly team meetings involving GPs, community nurses, and social workers aimed to define shared plans for patients.
- Joint home visits and cases/individual evaluations involving the team and, whenever necessary, other healthcare and social services.
- Structured and regular team meetings—called “*tavolo della complessità”* (complexity roundtable)—between members of the multiprofessional team to take charge and care of patients with complex needs.
- Collaboration with third-sector associations operating in the surrounding area.
- Engagement and networking with representatives of the community to organize and co-design the spaces of the structures and to define priorities and ways to collaborate in health and social services.
- Educational projects for master students and residents in different disciplines—mostly in public health, primary care, nursing, architecture, and urbanism. In these activities, HPs are involved in lessons using both classic and new interactive educational methodologies.

These collaborative practices require continuous remodeling to adapt to the changing context and evolving needs.

The new concept of HoC embeds these principles and aims to enhance community resources and participation. A cultural organizational change is required to realize this mandate and to support new and modified working practices concerning health promotion, care and relationships among HPs, social workers, and community actors. These changes must embrace the theoretical principles of c-PHC and must adapt to the health needs of the population in specific geographic and social contexts.

## 3. Discussion

### 3.1. Methods: how to define the model of HoC as the evolution of HoH

In our local context, the willingness and interest expressed by young HPs working in the HoHs have led to the definition of a Participatory Action Research (PAR) process (21) supported by the university and LHD. This approach represents the methodology within which the change takes place, in an ever-evolving process as the construction of the HoC itself is. Young HPs, HPs working in the HoHs, researchers, and students constitute a research group focused on overcoming the obstacles toward the flourishing of new community health services and delving into the underlying causes and dynamics. The group has strong motivation. Some HPs and public health researchers were students when the project was launched. This lets them pursue reflection actions and apply learned approaches and working practices (22). The focus is not on the results of each single research step in the management of care but also on the continuity of the process itself in reorganizing primary care services as community-oriented. Reflection on working practices encompasses specific instruments of conflict mediation and reflection and observation spaces during the daily current services, together with research methodologies to reflect on and learn from these practices, analyze and discuss them, and then define improvement actions (23, 24).

Along the process, multiprofessional education and community-based interventions have been organized from the perspective of assuming a transformative role (25, 26).

Starting from these premises, within the process described, different levels of action must coexist to build this model.

- **The organizational level**: The LHD, including the HoCs (evolving from the HoH), must ensure the provision of healthcare services, take care of health needs, and adjust the services provided according to the evolving needs. LHDs and HoCs must adapt to new guidelines and regulatory changes, extending their roles to management, planning, prioritization, organization of activities, monitoring, and evaluation.
- **The experimentation of new models of care**: It is associated with the demand to better answer health needs. Changing working practices toward more integrated actions requires organizational modification (27), team reflexivity on team roles and processes, and the definition of tools for monitoring and evaluating teamwork effectiveness and quality (28, 29). At the same time, the engagement of HPs is required to make this organizational culture change in a sustainable and participatory way (30).
- **The research level**: It complements the whole process by means of several research methods (research-action process, quantitative, and qualitative methodology) and different research issues: context and health needs deepening and analysis, community engagement, analysis of HPs' needs and perceptions of their work, and developing the enabling factors toward multiprofessional and collaborative practices. The research process supports the definition of monitoring and evaluating tools for implementing the new practices. It involves researchers, HPs, and students.

The three levels should coexist and be coherent in the theoretical, ethical, and political framework of c-PHC. Bottom-up experiences could not only trigger those changes and innovative working practices but also guide a research process embedded in making them sustainable and acceptable (31).

### 3.2. Lessons learned and sustainability of the organizational process

The following elements are relevant to make the described organizational changes sustainable:

- **Development of the ongoing process:** Within the research-action process, analysis, monitoring, and continuous evaluation, with the participation of HPs and other actors involved, should be an essential condition to combine the elements of the HoCs model (evolving from the HoHs), c-PHC oriented and to make it feasible with the characteristics of the contexts.

° It includes the management of the macro-process and the different phases and the process direction toward the principles to which it aims to be implemented:
Moreover, it should include a reflection toward:
° Daily working practices: the implementation of collaborative integrated work needs the support of experimental working practices and integration between different levels (top management, middle management, and professionals in the field). Identification and discussion of HPs' problems is the first step to finding solutions and making the changes happen (27).
° Ethical implications of working practices and organizational model: a continuous reflection on whether, or not, this kind of organization is able to answer complex health needs and reduce health inequalities and barriers to access to care (18, 32, 33);
° Research: the research process involves and is part of an existing context, and consequently, it modifies relationships and dynamics both inside the HoC and in the community (34).

- **Caring for the working relationships among HPs and services**: Within the institutional mandate and in the context of HoC, working practices of HPs and social workers can launch organizational changes to improve teamwork and relationships, with a shared willingness to build them (18, 28, 35–37). The efficacy of the multiprofessional teams benefits from a dedicated time frame and physical spaces to reflect (29) and discuss the status quo, obstacles, and solutions (23, 24).

As a consequence, **the availability of specific competencies is also required**. Collaborative team meetings benefit from the presence of professionals with capabilities in leading groups (38–40). They could facilitate goal definition and how to get it, but also the communication process, and the achievement of shared decisions through conflict mediation, a shared “language,” and values. Reflexivity and periodic assessment of the group process are required (41–43).

- **Appropriate research methodology together with a “permeable” multidisciplinary research group**: Research design has to match the experiences and needs of the HPs involved, students, and other research actors in defining priorities and reflecting on working practices. Both qualitative and quantitative methodologies using a participative approach are required (44).
- **Education**: Designing IPE and CBE programs within a real context is central to implementing an HoC based on the c-PHC framework with the following specific learning outcomes (20, 25): foster new collaborative models in PC and between HP relationships; define tools and ways to assess and monitor the impact of the new model; share and disseminate c-PHC culture; and, as mentioned above, assess, evaluate, and monitor the activity, improve the quality of health services and relationships between different HPs and comply with the most recent scientific evidence. This goal benefits from interprofessional group meetings that involve HPs from different disciplines, researchers, students, and members of the community.

## 4. Conclusion

In our context, HoCs play the role of a permanent laboratory of experimentation, in which HPs interact with researchers, students, and future HPs, as well as the community and surrounding environment (citizens, policymakers, third sector associations, etc.), to overcome obstacles toward the flourishing of community health services.

This process shall embody different levels—institutional, relational, and interpersonal—and areas—education, management, organization, and daily working practices—as well as specific research to support it. The simultaneous coexistence of these levels can be the key to transform the relationships between HPs and how they see themselves (17); students and trainees can experiment with a multidisciplinary learning space along with discussions with HPs from different disciplines and with community actors and also renewing work practice. Commitment and responsibility toward the community are the framework within which knowledge, skills, and techniques acquire meaning. The trigger and development of an ongoing process—within a context of primary care reorganization as one described—is desirable and applicable in any context in which organizational and cultural changes in working practices occur.

Future research calls for further evidence on the impact of this model on community health and HP satisfaction and on the sustainability of the process. Moreover, the comparison with similar experiences in other contexts could help identify enabling factors and obstacles to overcome and common lessons to share.

Considering the scientific evidence and ethical issues concerning primary care and the real inclusion of all stakeholders, the HoC becomes the place of practical implementation of integrated services that contribute to the improvement of community health.

## Data availability statement

The original contributions presented in the study are included in the article, further inquiries can be directed to the corresponding author.

## Author contributions

CM, GN, LB, GB, and MN contributed to the design of the work. GB, CM, and GN devised the project and the main conceptual ideas. CM and GN wrote the first draft of the manuscript. All authors provided critical feedback and commented the manuscript. All authors contributed to the article and approved the submitted version.
